# Effects of Seasonality and Pregnancy on Hair Loss and Regrowth in Rhesus Macaques

**DOI:** 10.3390/ani14050747

**Published:** 2024-02-28

**Authors:** Allison Heagerty, Rebecca A. Wales, Kristine Coleman

**Affiliations:** Animal Resources & Research Support, Oregon National Primate Research Center, Oregon Health & Science University, Beaverton, OR 97006, USAcolemank@ohsu.edu (K.C.)

**Keywords:** macaque, *Macaca mulatta*, alopecia, hair regrowth, anagen, telogen, pregnancy

## Abstract

**Simple Summary:**

Although alopecia is prevalent among captive rhesus macaques, its cause is not well understood. Poor coat quality may raise concerns because it can be a byproduct of conditions such as stress, autoimmune disease, hormonal imbalance, infection, or poor nutrition. Despite lack of consensus as to the cause(s) of alopecia, multiple studies in captive primates have found two commonalities: alopecia fluctuates seasonally, and pregnant females tend to have more alopecia than males or nonpregnant females. Most studies have focused on loss of hair, rather than if and when hair is regrown, but alopecia can result from disruption to any phase of the hair follicle’s cycle of shedding and regrowth. To better understand how season and pregnancy affect the hair follicle cycle and alopecia, we documented the severity of alopecia and the presence of hair regrowth in outdoor group-housed rhesus for one year. We found a seasonal pattern of alopecia and regrowth in all animals, and that females in their third trimester showed less regrowth, which prevented a decrease in alopecia. Regrowth for females resumed on average 1–2 months postpartum. Hair shedding and regrowth follows a seasonal pattern in rhesus, and conditions in late-term pregnancy suppress hair regrowth into early postpartum.

**Abstract:**

Several studies have examined the etiology of alopecia, or hair loss, in rhesus macaques. While outcomes differ across studies, some commonalities have emerged. Females, particularly pregnant females, show more alopecia than males, and alopecia follows a seasonal pattern. Much research has explored causes of hair loss; however, alopecia can result from lack of hair growth in addition to hair loss. To better understand how sex, reproductive state, and season affect alopecia, we followed 241 rhesus macaques (*Macaca mulatta*) in outdoor breeding groups over one year, recording both alopecia severity and presence of hair regrowth. We found that both alopecia and hair regrowth followed a seasonal pattern; alopecia was highest in spring and lowest in late summer, while regrowth started in spring and peaked in late summer. Reproductive state also correlated with both alopecia and hair growth. Females in their third trimester had the highest average level of alopecia and the lowest amount of hair regrowth. Regrowth resumed postpartum, regardless of whether females were rearing an infant. Results indicate that the seasonal pattern of alopecia is due in part to the seasonal limitations on hair regrowth, and that breeding, which also occurs seasonally in rhesus macaques, may further suppress hair regrowth.

## 1. Introduction

Alopecia, or the loss or absence of hair, is prevalent among captive populations of macaques (*Macaca* spp.) and other nonhuman primates (NHPs). Studies have investigated an array of potential contributors to alopecia in NHPs, including stress [1,2,3,4,5,6], nutritional deficiencies [7], chronic inflammation [8], behavior, such as self-epilation or hair pulling by social partners [9,10,11], temperament [12], and facility of origin [2,13,14]. While no one single cause has been identified, there are some correlates consistently found by surveys of alopecia within NHP populations. First, alopecia has been associated with sex and reproductive status in macaques. Several studies have found that female monkeys, especially pregnant females, typically show higher degrees of hair loss than males [3,6,15,16,17,18,19]. Dettmer and colleagues [17] additionally found that among pregnant females, hair cortisol was positively correlated with alopecia and infant birth weight and growth, and Lutz [18] found that among baboons (*Papio hamadryas* spp.), dams with female infants had more alopecia on average than those rearing male infants during the postpartum period, but not during pregnancy. These studies suggest a role for stress and/or energetic demands in alopecia, during and after pregnancy.

Additionally, longitudinal studies of outdoor-housed rhesus macaques consistently show a seasonal pattern, with alopecia being highest in spring and lowest in late summer and early fall [3,6,15,20,21,22]. However, as rhesus macaques are seasonal breeders (breeding typically in fall and early winter and birthing in spring and early summer), it can be difficult to separate effects of time of year from effects of reproduction. An early survey of hair “molt”, or shedding, on Cayo Santiago documented the seasonal shedding and regrowth patterns for two groups of rhesus macaques [23]. The authors found that molt typically began in late spring and completed in mid-summer for males and females without infants. Females with infants started and ended molt approximately one month later.

When not due to hair pulling behavior (either self-inflicted or by conspecifics), alopecia can be caused by an alteration to hair follicle cycles. The hair follicle cycle has three main phases: anagen, when the hair shaft is actively growing; catagen, when the hair follicle shrinks; and telogen, the resting phase. In a normal hair cycle, the old hair shaft begins to detach from the follicle and is shed in a process called teloptosis or exogen [24,25,26,27]. Teloptosis usually overlaps with early anagen as a new hair shaft is growing, but can occur at the end of telogen without anagen having begun [25]. In humans, head hair follicles usually cycle asynchronously (i.e., follicles are not all in the same phase of the cycle at the same time), whereas the hair follicles of most other mammals cycle in synchrony [24], thus resulting in a conspicuous molt and regrowth as documented by Vessey and Morrison [23].

The alteration of follicular cycles leading to alopecia can be seen in humans during and after pregnancy or following a stressful event. Telogen effluvium is an umbrella term for conditions characterized by a period of dramatically increased hair loss, usually occurring 3–4 months after a stressful life event or major illness [24,27,28]. Postpartum telogen effluvium refers to the condition when the triggering event is childbirth. Some women experience increased hair loss in the 2–6 months following childbirth due, in part, to a large portion of the hair follicles synchronizing in telogen, and consequently undergoing teloptosis all at once [24,26,27,29,30]. Researchers do not yet know why some people experience telogen effluvium and others do not, or the mechanism by which hair follicles become synchronized in humans.

Other forms of alopecia can result from disruptions at various points in the hair follicle cycle. For example, researchers have experimentally altered the hair cycle in mice by inducing stress. Aoki and colleagues [31], and later Katayama and colleagues [32], found that a foot shock prevented hair regrowth by prolonging telogen and delaying anagen in mice. Other researchers, using noise to induce stress in mice, found that hair follicles prematurely terminated the anagen phase and entered catagen [33], similar to the hair follicles of people with alopecia areata (hair loss caused by an autoimmune condition) [34]. Thus, in addition to alopecia caused by hair pulling or excessive shedding (in the case of telogen effluvium), alopecia can also result from normal shedding accompanied by delayed or premature termination of regrowth. 

Studies of alopecia in NHPs primarily focus on hair loss, or teloptosis. Fewer studies have specifically examined regrowth, or anagen, but both hair loss and disruptions to regrowth could play a role in alopecia. For example, several studies have found that alopecia increases with pregnancy [3,6,15,16,17,18,19], which could result from these animals actively losing hair, not regrowing hair, or a combination of both. To better understand how season and pregnancy may interact to affect alopecia in terms of the hair follicle cycle, we documented the presence and severity of alopecia, and for females, pregnancy status, of breeding-age rhesus living in outdoor social groups for one year. We additionally recorded hair regrowth, as a first step in understanding the role that inhibition of hair growth may play in alopecia. Based on prior research, we predicted that pregnant females would show more alopecia than males or nonpregnant females, and that alopecia severity would fluctuate with time of year, with the highest levels of alopecia corresponding to the birthing season in spring. As with alopecia, we expected regrowth to follow a seasonal pattern, as prior studies have found, with regrowth primarily occurring in summer [23].

## 2. Materials and Methods

### 2.1. Subjects and Housing

Subjects were 241 rhesus macaques (*Macaca mulatta*) housed in eight outdoor enclosures (described below) at the Oregon National Primate Research Center (ONPRC) from December 2014 through November 2015. All subjects were born and reared at the ONPRC. One group was disbanded 7 months into the study (June 2015) due to hierarchy instability, and the remaining seven groups were studied the entire 12 months. Groups ranged in size from 43 to 67 animals (mean = 55, SD = 8), and were comprised of one older adult (>9 years) male, multiple adult (>3 years) females, and infants and juveniles. Most groups also had young adult (3–5 years) males (Table 1). The mean age of breeding-age animals (≥3 years) was 7.4 +/− SD 3.19 years for females, and 9.7 +/− SD 4.86 years for males. Subjects were individually identifiable by tattooed identification numbers, characteristic facial features, body shapes, and coat color. 

Subjects were housed in outdoor, environmentally controlled “sheltered housing” units. Each unit had an area of approximately 130 square meters subdivided into three rooms. Two openings, approximately 0.5 m by 0.6 m, in each of the two dividing walls allowed animals to move freely between rooms. Three of the four exterior walls were comprised of a low cement wall with wire mesh extending to a mesh ceiling, below a translucent plastic roof. The fourth wall, and the walls subdividing rooms, were cement from floor to mesh ceiling, or cement covered with marine board. Supplemental heat was provided during cold temperatures in the form of radiant heating above the center room, and hydronic radiant floors in all rooms. During warmer months, cooling was achieved by overhead misters, fans, pools, and sprinklers. Enclosure enrichment included climbing structures, fixed perches, and a variety of swings and toys. Fresh produce or grain was distributed daily to encourage foraging. 5000—Fiber-Balance Monkey Diet, Lab Diet (Land O’ Lakes, Inc., Arden Hills, MN, USA) was fed twice daily ad libitum and water was continuously accessible from six lixit faucets (QC Supply, Schuyler, NE, USA) located throughout each enclosure. The ONPRC is accredited by AAALAC, International, and this study was approved by the ONPRC Institutional Animal Care and Use Committee. 

### 2.2. Alopecia and Hair Regrowth Assessment

One of two raters (AH and RAW) evaluated alopecia and hair regrowth on all animals 3 years old and older in each group monthly. Raters were reliable at >85% agreement using the Behavioral Management Consortium’s alopecia scoring method, designed to estimate the amount of the body affected by alopecia [35]. An area was considered affected by alopecia if skin was visible where it would not be visible with a full coat. Using this method, we estimated the overall percentage of the body affected by alopecia, then converted the percentage to an ordinal score from 0 (<1% alopecia) to 5 (≥75% affected) (see Table 2). Raters also noted the presence or absence of newly grown hair (i.e., regrowth) at the time of alopecia scoring. Since growing hair takes time to reach its full length, an area with regrowth could initially also have been counted as still being affected by alopecia. All group members were sedated for physical exams quarterly for a different study, at which time the raters assessed alopecia and regrowth while animals were under sedation. In months without sedated physical exams, alopecia and regrowth were scored from cage-side observations of the animals. 

### 2.3. Reproductive Status

For each alopecia scoring timepoint, we characterized the reproductive state of females as: nonpregnant; first, second, or third trimester of pregnancy; or postpartum (for the 6 months post-delivery). For the postpartum period we also recorded whether they were rearing an infant. The ONPRC outdoor rhesus colony follows a seasonal pattern of breeding and birthing, with breeding occurring primarily from late September through January, and most births occurring from March through July. A trained health technician recorded any new births each morning during daily health observations of animals. We determined trimester of pregnancy at each alopecia scoring retroactively by subtracting the scoring date from the day of parturition, assuming a gestation length of 165 days and trimesters of 55 days each [36]. In the case of stillborn infants, we used the gestation age estimated by a clinician during physical exam of the dam prior to parturition, and fetal age estimated at necropsy to determine the reproductive state of those females at alopecia scoring timepoints. Additionally, females were palpated to determine if they were pregnant or nonpregnant by a clinician during quarterly physical exams. Still, it is possible, though likely uncommon, that some pregnancies went undetected if spontaneous abortion occurred early in gestation. 

### 2.4. Data Analysis

We converted the ordinal alopecia scores to z-scores to obtain a normal distribution. To assess the relationship between pregnancy and alopecia z-scores, we used generalized linear mixed model regression with a Gaussian distribution. We tested the independent variables of month and reproductive state. Month was month of the study, such that the first month of data collection (December 2014) was month 1. Because alopecia is expected to follow an annual cycle, and therefore have a curvilinear relationship to study month, we included a quadratic (i.e., squared) term for study month as well. Reproductive state encapsulated sex and reproductive status of females, so that animals were categorized as one of the following at each alopecia scoring: male; nonpregnant female; first, second, or third trimester pregnant; postpartum rearing an infant; or postpartum without an infant. We controlled for age by including this as an independent variable, and included individual animal ID nested within a social group identifier as a random effect. 

A binary outcome of regrowth presence or absence was modeled using generalized linear mixed model regression with a binomial distribution. The independent fixed effect and random effect variables tested were the same as listed above for the analysis of alopecia z-scores.

We used Akaike Information Criteria (AIC) scores to evaluate regression models. AIC scores prioritize use of the minimum number of independent variables to fit the data, with lower scores indicating better fit. Differences of two or more AIC points are considered meaningful [37]. We assessed estimates of independent variables within regression models using a criterion of alpha = 0.05.

To test the effect of nursing an infant on hair regrowth, we performed a post-hoc two-tailed t-test on the number of days between parturition and first observation of hair regrowth for females rearing an infant and those not rearing an infant. We determined these data to be normally distributed by a Q-Q plot and a Shapiro–Wilk Test (W = 0.99, *p* = 0.25).

We used R version 4.0.3 for all analyses [38].

## 3. Results

### 3.1. Summary of Data Collected

Table 3 shows the number of subjects per reproductive status category scored for alopecia and presence of hair regrowth during each study month. The number of males scored increases in May and June due to animals reaching 3 years of age. Because rhesus macaques are seasonal breeders, not all categories are represented each month. Of the 208 female subjects, 94 gave birth during the study. Of those, 63 reared infants for at least 6 months, five reared infants to less than 6 months due to infant illness, and 26 did not rear infants due either to infants being removed for research or clinical reasons, or infants being stillborn. 

### 3.2. Alopecia Severity

**Figure 1:**
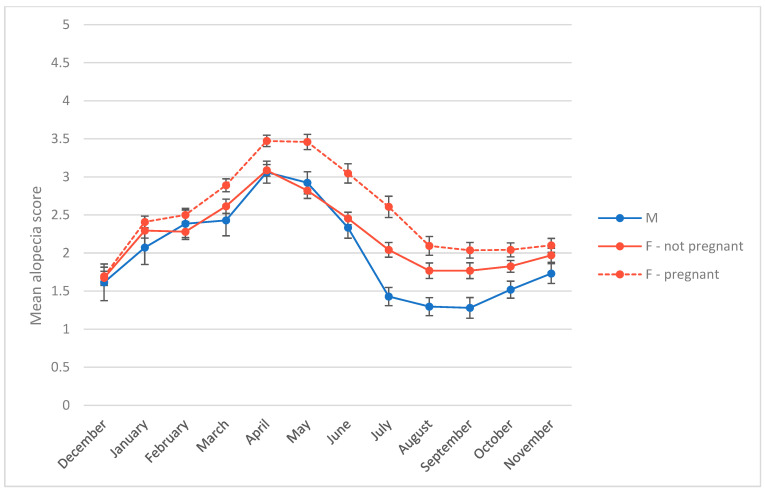
Mean alopecia scores by month for males, females that had a detectable pregnancy during the study, and females that did not. Error bars represent standard error.

**Figure 2:**
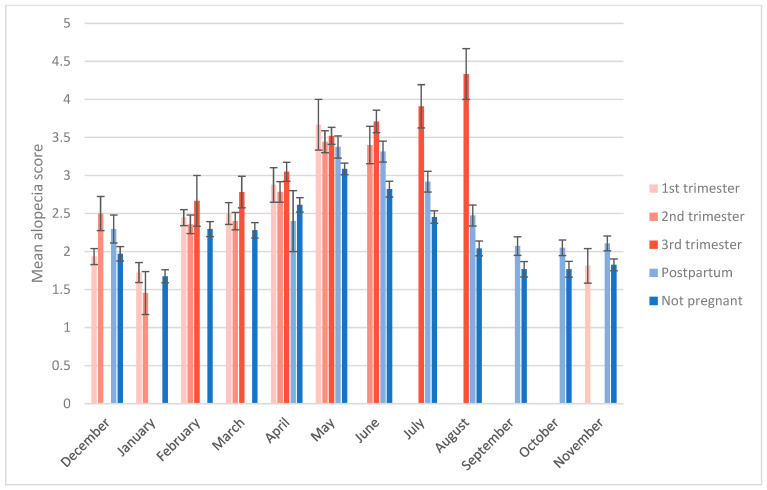
Mean alopecia scores by month for females of various reproductive states. Error bars represent standard error. Six timepoints were only represented by one animal (see Table 3) and were thus omitted from this graph because of the inability to calculate standard errors.

The first objective of this study was to measure the relationship between alopecia scores, study month, sex, and reproductive state in our breeding colony. The best regression model for alopecia z-scores had an AIC score 50 points lower than the next-best model (Table 4). This model contained main effects of study month, study month squared, and reproductive state, as well as an interaction between study month and reproductive state. The intercept represents nonpregnant females (B = −0.31, *p* < 0.01). The significant study month terms (study month B = 0.24, *p* < 0.001; study month squared B = −0.02, *p* < 0.001) indicate a curvilinear relationship between month and alopecia. Among our subjects, alopecia peaked in April and May, and was lowest in August and September (Figure 1). Main effects of being pregnant (first, second, or third trimester) were not significant, but interacted significantly with study month such that alopecia increased with gestation as months progressed, with females in their third trimester being most affected in July and August (Figure 2) (study month × 1st trimester B = 0.03, *p* = 0.04; study month × 2nd trimester B = 0.06, *p* = 0.03; study month × 3rd trimester B = 0.12, *p* < 0.01). Being postpartum had a significant main effect on alopecia (postpartum B = 1.75, *p* < 0.001; postpartum rearing an infant B = 1.38, *p* < 0.001), but this effect interacted with study month to decrease as months passed (study month x postpartum B = −0.21, *p* < 0.001; study month x postpartum rearing infant B = −0.13, *p* < 0.001), and postpartum females had lower alopecia scores than pregnant females, particularly in July and August (Figure 2). Male alopecia scores were not significantly different on average from nonpregnant females, but being male also interacted with study month to decrease alopecia over time (study month x male B = −0.05, *p* < 0.01). Thus, on average, females who had been pregnant showed the highest alopecia scores throughout the year, in particular third trimester females in summer, whereas females who did not have a detectable pregnancy and males showed similarly low levels of alopecia, except in July through October when male scores were lowest (Figure 1 and Figure 2).

### 3.3. Presence of Hair Regrowth

**Figure 3:**
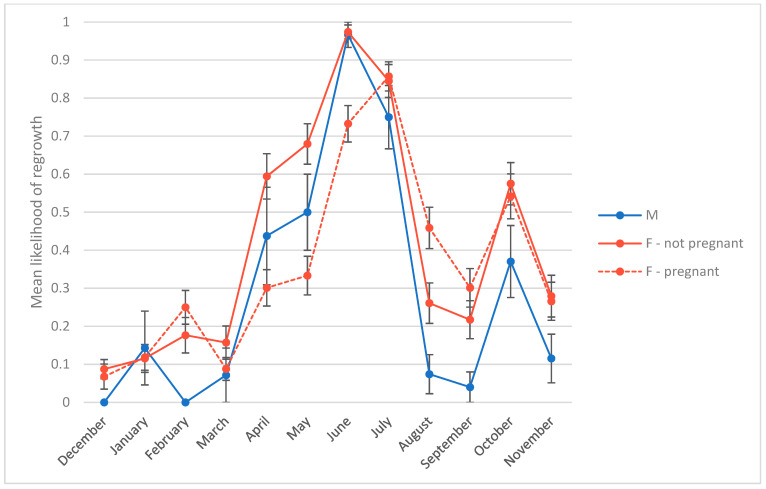
Mean likelihood of hair regrowth by month for males, females that had a detectable pregnancy during the study, and females that did not. Error bars represent standard error.

**Figure 4:**
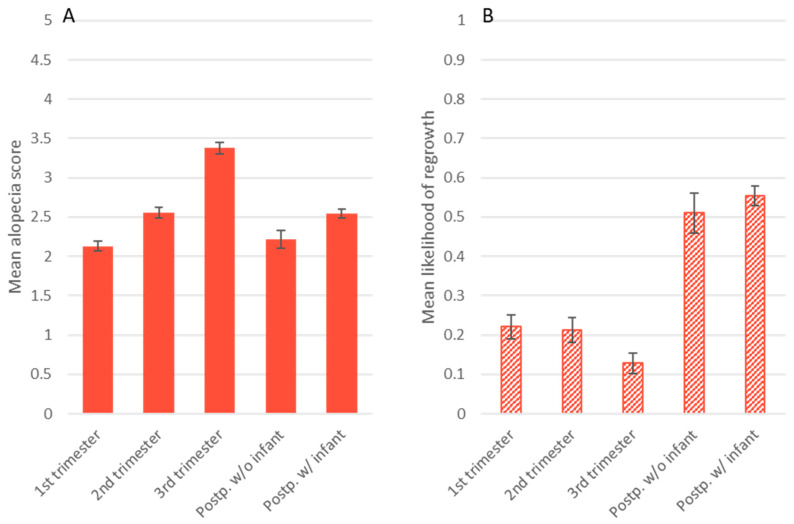
Mean alopecia score (**A**) and mean likelihood of observing hair regrowth (**B**) of females who had a detectable pregnancy in the first, second, or third trimester, postpartum without an infant (Postp. w/o infant) or postpartum with an infant (Postp. w/infant) stages. Error bars represent standard error.

The second objective of this study was to test whether hair regrowth is related to time of year, sex, age, and/or reproductive state. The best model for presence of hair regrowth had an AIC score 82 points lower than the next-best model. As with the model for alopecia score, the regrowth model contained the main effects of study month, study month squared, and reproductive state (Table 5). As in the alopecia model, study month had a significant and curvilinear effect (study month B = 1.43, *p* < 0.001; study month squared B = −0.10, *p* < 0.001). Among our subjects, the likelihood of regrowth increased until its peak in June and July, before declining again. The data showed an additional peak in October that the regression model did not account for (Figure 3). On average, males were less likely to show regrowth than nonpregnant females (B = −0.63, *p* < 0.001) (Figure 3). Likelihood of regrowth for females in their first trimester was not significantly different from nonpregnant females, represented by the intercept (intercept B = −4.52, *p* < 0.001). However, females later in gestation were less likely to show regrowth, in particular females in their third trimester (2nd trimester B = −0.53, *p* = 0.02; 3rd trimester B = −2.15, *p* < 0.001) (Figure 4). Postpartum, likelihood of showing regrowth returned to levels similar to those of nonpregnant females, regardless of whether the female was nursing an infant or not (postpartum B = −0.36, ns; postpartum rearing infant B = −0.03, ns) (Figure 4). Thus, it appears as though hair regrowth is suppressed for females in their third trimester, but regrowth resumes postpartum. This delay in regrowth for pregnant females means that on average, breeding females reach their peak of regrowth 1 month later (in July) than males and nonpregnant females. By October, breeding females show regrowth at rates on par with nonbreeding females (Figure 3).

### 3.4. Time to Regrow Hair Postpartum

The number of days between infant delivery and the first recorded scoring of hair regrowth ranged from 0 to 82 days, with a median of 40 days. This aligned with our anecdotal observation that regrowth was usually observable at the first or second postpartum scoring, in other words, within 4–8 weeks following parturition. A two-tailed *t*-test of days to regrow hair post-birth for females with and without infants did not indicate a significant difference (t_(72)_= −1.19, *p* = 0.24). It is important to note that because we assessed regrowth once per month and not daily, our data on number of days between infant delivery and regrowth are approximate. 

**Figure 5:**
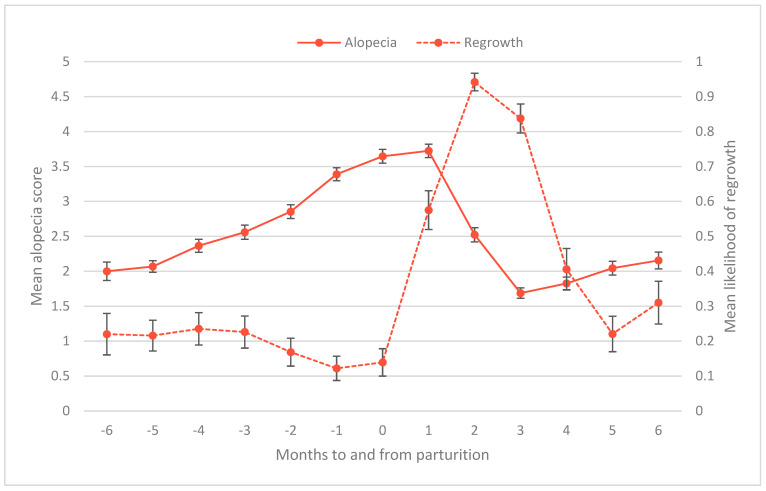
Mean alopecia score (left vertical axis) and mean likelihood of regrowth (right vertical axis) for females with a detectable pregnancy in relation to months to and from parturition. Error bars represent standard error.

Figure 5 shows the effect of gestation on regrowth (y-axis on the right) and subsequently on alopecia scores (y-axis on the left) in relation to months to and from parturition. As females near infant delivery, regrowth declines and alopecia increases. In the two months after giving birth, regrowth increases sharply, corresponding to a steep decline in alopecia scores. As regrowth decreases again over time, after four months postpartum, alopecia scores begin to rise again (Figure 5).

## 4. Discussion 

The degree to which alopecia indicates a welfare problem depends on the underlying causes of the hair loss. Alopecia that results from normal biological processes, such as aging or reproduction, may not indicate compromised well-being. Conversely, if an animal undergoes an acute loss of hair after a stressful event (e.g., a housing change), that is probably a case that should be addressed. See Novak and Meyer (2009) for a discussion of the welfare implications of alopecia and recommended approaches for evaluating and treating animals with the condition [5]. Because of the potential welfare implications associated with alopecia, several researchers have examined potential causes underlying hair loss in rhesus macaques and other NHP species. While studies have found a variety of factors that correlate with alopecia, implicating a multimodal nature to the condition, there have been some trends consistently identified. Multiple studies in macaques and other primate species have found that pregnancy influences alopecia such that pregnant females tend to have less hair than males or nonpregnant females [3,6,15,16,17,18,19]. Similarly, studies have found that alopecia severity fluctuates throughout the year, with monkeys typically having the least hair in spring and the most in summer [3,6,15,20,21,22]. Given that rhesus macaques are seasonal breeders, with spring corresponding to the peak of the birth season, the degree to which time of year, as opposed to female reproductive status, influences alopecia is difficult to separate. 

At the follicular level, alopecia can result from hair being forcibly removed by the animal itself or by a conspecific (i.e., hair pulling or plucking), or from a disruption to the normal hair growth cycle of anagen (growth phase), catagen (follicle shrinkage phase), telogen (resting phase), and teloptosis (shedding). For example, research in humans has shown that alopecia can be caused by synchronized teloptosis to cause telogen effluvium, a condition seen in postpartum women or following a major stressor [24,27,28,29]. Studies in mice have shown that intermittent stress can either prolong telogen, and therefore delay the subsequent anagen stage, or terminate anagen prematurely. Both effects could result in alopecia by preventing regrowth if teloptosis, i.e., shedding, has already occurred [32,33]. Understanding whether the hair follicle is cycling normally might provide insight into causes of alopecia. As a first step, we examined both hair loss and regrowth over the course of a year in group-housed rhesus macaques.

Similar to other studies [3,6,15,20,21,22,23], we found a seasonal pattern to alopecia in our subjects, although a multi-year study tracking individuals over time would be needed to conclude that the pattern we observed is truly seasonal. Alopecia increased in the winter months, peaked in spring, and decreased in the summer months for all sex and reproductive categories. We also found a seasonal pattern to regrowth among all subjects, with regrowth starting in early spring and peaking in late spring/early summer, and showing another, smaller peak in fall. However, there did seem to be an interaction between time of year and reproductive status. During winter, alopecia increased at a similar trajectory for all sex and reproductive groups until spring, at which point many females reached their third trimester of pregnancy, and seasonal regrowth began for males and nonpregnant females. This delayed time to regrow hair for pregnant females seems to have resulted in higher levels of alopecia for breeding females than males or nonpregnant females, due to continued hair loss with lower levels of regrowth. This finding suggests that the hair follicles of third trimester females are in prolonged telogen, preventing anagen, although histological analysis of hair follicles are needed to confirm this hypothesis. 

The underlying mechanism by which reproduction affects the hair follicle cycle is unclear. It has been suggested that the energy demands of lactation influence alopecia. Dettmer and colleagues [17] and Lutz [18] found that alopecia was positively correlated with indices of maternal investment in offspring in rhesus macaques and baboons, respectively. Additionally, several hormones and other chemical messengers involved in pregnancy have been shown to influence the hair follicle cycle, such as estrogen and prolactin, and stress-mediated chemicals such as glucocorticoids and substance P [29,33,39]. In our study, once females had their infant, their likelihood of showing regrowth returned to levels similar to that of nonpregnant females by the first or second subsequent monthly scoring. Further, whether or not a female was rearing an infant did not affect time to regrow hair. These results may indicate that hormones, not energy demands, are suppressing hair growth during pregnancy, since lactation is more energetically costly than gestation [40]. However, our results regarding lactation and hair regrowth should be taken with caution, as there are many factors that influence an individual female’s energy output during lactation that we did not control for, including infant sex, maternal rank, and parity [41]. Further research is needed to determine the role these factors play in the relationship between alopecia and lactation. 

Another approach to understanding the connection between pregnancy and hair loss is to compare related species. For instance, rhesus and Japanese macaques (*M. fuscata*) are both seasonal breeders and show sex differences with respect to alopecia, with females typically more affected than males [22]. However, in a study comparing alopecia among two aseasonal breeders—pigtail (*M. nemestrina*) and cynomolgus (*M. fascicularis*) macaques—and rhesus macaques, the authors found that while the aseasonal breeders did show a seasonal pattern of alopecia, there were no differences in severity between males and females [20]. Future research examining the biological differences between species and how they may affect hair loss and regrowth could help us untangle the effects of reproduction and time of year on alopecia. 

This study is among the first to look at alopecia and regrowth in outdoor-housed populations of rhesus macaques. While more work is needed to determine what drives the seasonal pattern of alopecia, it seems as though the seasonal pattern of hair regrowth may be a large contributor. Our results show that hair growth is not constant throughout the year for outdoor-housed rhesus macaques, but rather depends on time of year and reproductive status of the animal. Because hair regrowth is not consistent throughout the year, alopecia increases when regrowth rates are low, and decreases when regrowth surges. This finding has implications for potential treatments for rhesus macaques with hair loss. Alopecic monkeys are often provided with a variety of enrichment items, such as grooming boards covered with trail mix, with varied levels of success [5]. Our results suggest there may be normal physiological reasons for coat quality not to improve immediately.

## 5. Conclusions

We found a seasonal pattern of both alopecia severity and hair regrowth among our outdoor breeding groups of rhesus macaques. Alopecia severity increased during times of year without regrowth (winter and early spring), and decreased in late spring and summer when regrowth occurred. These patterns were present among males and nonpregnant females, and suggest that the hair follicle cycle disruption is not the cause of alopecia in these demographic groups. However, females who were pregnant during the study had higher levels of alopecia, which seemed to be driven by inhibited hair regrowth during the third trimester of pregnancy. For these females, regrowth began within 1–2 months postpartum. Alopecia in these females may be exacerbated by a disruption to the hair follicle cycle which is preventing anagen. There was no difference in when hair regrowth began for females who had been pregnant and were rearing an infant and those who had been pregnant but were not rearing an infant, suggesting that hormones, rather than energy expenditure, inhibits regrowth for pregnant females. 

## Figures and Tables

**Table 1:** Summary of demographics of study groups. Age categories reflect ages at the conclusion of the study. Group 1 was disbanded after 7 months of study in June 2015 due to social instability.

Group	1	2	3	4	5	6	7	8	Total
Males > 3 years	3	6	7	1	3	2	6	5	33
Females > 3 years	25	29	17	34	23	23	20	37	208
Males & females < 3 years	20	26	19	32	29	26	24	21	197
Total	48	61	43	67	55	51	50	63	438

**Table 2:** Alopecia scoring system based on the percent of the body showing alopecia.

Percent of Body Affected by Alopecia	Alopecia Score
0	0
1–9	1
10–24	2
25–50	3
51–74	4
75–100	5

**Table 3:** Number of animals scored of each sex and reproductive category each month. One female who was postpartum in December–February had given birth in the prior birthing season in 2014. The first birth during study occurred in February 2015.

		Females					
Month	Male	Nonpregnant	1st Trimester	2nd Trimester	3rd Trimester	Postpartum w/o Infant	Postpartum w/Infant
December	13	126	47	11			1
January	14	78	47	42	3		1
February	13	68	22	50	23		1
March	14	70	8	37	41		5
April	16	69	3	18	48	3	21
May	26	78		5	31	13	38
June	30	77		1	11	16	58
July	28	71			6	16	62
August	27	69			1	18	66
September	25	69	1			18	64
October	27	80	16			10	46
November	26	68	46	6		2	25

**Table 4:** Gaussian regression model estimates for alopecia z-scores. The intercept represents nonpregnant females.

	Estimate	Std. Error	*p*-Value (* Indicates Value < 0.05)
Intercept	−0.31	0.10	<0.01 *
Study month	0.24	0.02	<0.001 *
Study month squared	−0.02	<0.001	<0.001 *
1st trimester pregnant	−0.04	0.10	0.71
2nd trimester pregnant	0.07	0.13	0.57
3rd trimester pregnant	0.30	0.23	0.18
Male	0.18	0.16	0.26
Postpartum without infant	1.75	0.37	<0.001 *
Postpartum with infant	1.38	0.19	<0.001 *
Study month × 1st trimester pregnant	0.03	0.01	0.04 *
Study month × 2nd trimester pregnant	0.06	0.03	0.03 *
Study month × 3rd trimester pregnant	0.12	0.04	<0.01 *
Study month × male	−0.05	0.02	<0.01 *
Study month × postpartum without infant	−0.21	0.04	<0.001 *
Study month × postpartum rearing infant	−0.13	0.02	<0.001 *

**Table 5:** Binomial regression model estimates for likelihood of showing hair regrowth at time of alopecia scoring. The intercept represents nonpregnant females.

	Estimate	Std. Error	*p*-Value (* Indicates Value < 0.05)
Intercept	−4.52	0.31	<0.001 *
Study month	1.43	0.09	<0.001 *
Study month squared	−0.10	0.07	<0.001 *
1st trimester pregnant	0.23	0.22	0.31
2nd trimester pregnant	−0.53	0.24	0.02 *
3rd trimester pregnant	−2.15	0.27	<0.001 *
Male	−0.63	0.19	<0.001 *
Postpartum without infant	−0.36	0.25	0.15
Postpartum with infant	−0.06	0.15	0.69

## Data Availability

The data presented in this study are available by request from the corresponding author.
